# Sensors on the Move: Onboard Camera-Based Real-Time Traffic Alerts Paving the Way for Cooperative Roads

**DOI:** 10.3390/s21041254

**Published:** 2021-02-10

**Authors:** Olatz Iparraguirre, Aiert Amundarain, Alfonso Brazalez, Diego Borro

**Affiliations:** 1 CEIT-Basque Research and Technology Alliance (BRTA), Manuel Lardizabal 15, 20018 Donostia/San Sebastián, Spain; aamundarain@ceit.es (A.A.); abrazalez@ceit.es (A.B.); dborro@ceit.es (D.B.); 2 Universidad de Navarra, Tecnun, Manuel Lardizabal 13, 20018 Donostia/San Sebastián, Spain

**Keywords:** Cooperative Intelligent Transport Systems (C-ITS), traffic sign recognition, fog detection, intelligent roads, V2X communications, road maintenance

## Abstract

European road safety has improved greatly in recent decades. However, the current numbers are still far away to reach the European Commission’s road safety targets. In this context, Cooperative Intelligent Transport Systems (C-ITS) are expected to significantly improve road safety, traffic efficiency and comfort of driving, by helping the driver to make better decisions and adapt to the traffic situation. This paper puts forward two vision-based applications for traffic sign recognition (TSR) and real-time weather alerts, such as for fog-banks. These modules will support operators in road infrastructure maintenance tasks as well as drivers, giving them valuable information via C-ITS messages. Different state-of-the-art methods are analysed using both publicly available datasets (GTSB) as well as our own image databases (Ceit-TSR and Ceit-Foggy). The selected models for TSR implementation are based on Aggregated Chanel Features (ACF) and Convolutional Neural Networks (CNN) that reach more than 90% accuracy in real time. Regarding fog detection, an image feature extraction method on different colour spaces is proposed to differentiate sunny, cloudy and foggy scenes, as well as its visibility level. Both applications are already running in an onboard probe vehicle system.

## 1. Introduction

No deaths and serious injuries on European roads by 2050. This is the goal established by the European Commission (EC). Meanwhile, EU road safety targets halving these numbers by 2030 [1]. The EU has seen a substantial decrease in road fatalities in the past, but these numbers have been stagnating in recent years. The latest research studies indicate that even with the lockdown due to the COVID-19 pandemic situation, deaths on the road did not decline by the same degree as traffic volume did [2].

To address this trend and meet the targets, the EC is committed to digital technologies. Future intelligent vehicles will interact with other vehicles and with the road infrastructure. This interaction is the domain of Cooperative Intelligent Systems (C-ITS) and is expected to significantly improve road safety, traffic efficiency, environmental performance and comfort driving, by helping the driver to make better decisions and adapt to the traffic situation [3]. Additionally, the EU road safety policy framework also focuses on infrastructure safety and the updating of some legislative measures. 

Over the last decades, the remarkable development in technology and the increasing digitalisation made enormous advancements in the intelligent vehicles field. Its advanced electronics and mechatronics, communications and sensors made current vehicles scale-up levels of driving automation. However, there is still a long way to go before we are faced with fully autonomous and cooperative roads. The perception of the environment, given its high complexity, represents a challenge for the Intelligent Transport Systems (ITS) scientific community and industry.

One main requirement for intelligent vehicles is that they need to be able to perceive and understand their surroundings in real time. For this aim, intelligent vehicles are usually fitted with multiple sensors to collect a large amount of data. The work presented in this paper was developed as part of an industrial project that means to use vehicles as sensors, especially in interurban areas, where there can be long stretches of road without supervision. The primary objective of this project is to detect road events that could be useful to enhance road safety, such as meteorological events or defects in the infrastructure. To respond to the wide variety of different situations and elements that can occur on the road, a multiple-sensor was developed. 

This probe vehicle system is installed in maintenance vehicles that cover the entire intercity road network of Bizkaia (Spain). It is capable of covering several weather situations by detecting different levels of rain and fog. Moreover, it has an ad-hoc black ice prediction system onboard. Furthermore, it incorporates a traffic signalling monitoring system as a support tool for the maintenance of the road infrastructure. However, some of these sensors are often limited and expensive. Therefore, this paper is focused on the functionalities that use cameras in conjunction with computer vision techniques: a fog detection module and Traffic Sign Detection (TSR) module. These solutions are considered very attractive since vision sensors have the potential to measure a multitude of parameters and their maintenance is simple and low cost. 

On one hand, the real-time traffic sign recognition (TSR) module presented in this paper helps to automatize the supervision of the traffic signs. The latest report from the Spanish Road Association reveals that 374,000 code signs need to be renewed, 72% of them with expired reflective material [4]. Infrastructure plays an important role in road safety, and so do inspections to check its condition and detect possible incidences. Therefore, this TSR module keeps an updated and exhaustive inventory of the whole interurban road. This way it could help the operator to update the database when a new sign is installed or removed from the road. However, the greatest potential of the module lies in the comparison with the inventory to mark signs that have not been detected, either because they are deteriorated by the passage of time, accidents, vandalism, etc., or hidden by the growth of trees or other objects. This work presents the recognition of vertical traffic signs in several weather and light conditions. 

On the other hand, the real-time fog detection module addresses a hazardous location warning system for bad weather conditions; that is, foggy scenarios. Fog is one of the most feared weather episodes for drivers, it could happen at any time of the year and last for miles and miles or just disappear after a few meters. However, this kind of weather-related information is usually given through variable messaging panels (VMS). These are often imprecise because they are very distant from each other along with the road network or even non-existent on some roads. Therefore, this onboard fog detection system could enhance road safety since it allows us to communicate this information in real-time and send a high precision warning to other users of the road. In this study, we will address the detection of fog scenes and the classification of its severity according to the visibility level.

The main contributions of this paper are (1) creation of a new database, Ceit-TSR, of 264 real-world images containing different weather and lighting conditions as well as other artefacts, such as occlusions, motion blurring and windshield dirtiness; (2) implementation of a real-time detector and classifier for traffic sign recognition in an onboard system for complex situations; (3) creation of a new database for fog detection purposes, which includes about 300 km driving images in different weather conditions and presents three different fog levels; and (4) combination of three different colour spaces (RGB, HSV and XYZ) to differentiate clear, cloudy and foggy weather, and its visibility level, for the implementation of a real-time onboard system. 

The rest of the paper is organized as follows. Section 2 and Section 3 present a brief revision of the most relevant related works and datasets of the field. Section 4 describes the applied methods for the implementation while Section 5 shows the results obtained. All these sections are structured in such a way as to separate the two applications mentioned above (TSR and fog detection). Finally, Section 6 concludes the paper by summarising the results with a global discussion focused on its application. 

## 2. Related Works

### 2.1. Traffic Sign Recognition

In recent years, there has been a lot of work related to the TSR field due to the development of machine learning methods and especially with the new deep learning techniques. Thus, there is plenty of literature and surveys around this topic. However, many methodologies got obsolete when large public databases, such as the German Traffic Sign Recognition (GTSRB) [5] and Detection (GTSDB) [6] appeared. Usually, in the literature, the TSR task is divided into two different phases: detection and classification. The first phase consists of identifying the presence of one or more signals and locating them in the image. The second step is to classify the signal according to its category and meaning. 

The detection has been mostly covered with model-based techniques using simple features, such as colours and shapes, and they have achieved high performance for the Region of Interest (ROI) extraction. Several works are more inclined to other learning methods, such as Adaboost with Haar-like features [7], SVM [8], Local Binary Patterns [9] and other non-grey features, such as Aggregate Channel Features (ACF) [10,11]. With the development of deep learning, CNN-based detectors have also appeared both to train simple features [12] and as well as end-to-end networks [13]. Regarding classification, deep learning is the method with the best performance in recent years [14]. Among the deep learning models, the convolutional neural networks (CNN) are the most popular techniques [15]. 

Although these learning methods mentioned above can achieve state-of-the-art results, most of them often need assistance to reach good accuracy and efficiency [16]. Many non-technical challenges jeopardise the performance of these algorithms, especially when the resolution of the images is low or there are different lighting conditions, blur, occlusions or other artefacts. Thus, it is hard to identify the best method to solve the detection problem [14]. Currently, the improvement is focused on solving both detection and classification phases together in one step [16]. Additionally, researchers are also trying to solve this issue in all kind of weather and light situations [17,18]. 

### 2.2. Fog Detection

In contrast to TSR methods, the field of fog detection has not been as well studied. There are only a few investigations in the last twenty years. These studies can be classified into two different approaches: The first one relies on the computation of the visibility range based on Koschimieder’s law [19]. The second approach is based on the extraction of image characteristics. Some studies are focused on black and white images, most of them based on the study of grey histograms [20]. However, Liu et al. [21] address this problem by applying HSV colour space thresholding, which seems to reduce the number of parametrization compared with methods base on grey-level histograms. 

## 3. Datasets

### 3.1. Existing Traffic Sign Recognition Datasets

The traffic sign recognition research field has seen increased attention in the last years; therefore, since 2013, many new large datasets have been publicly available. This has allowed many comparative studies that have helped to improve existing algorithms. However, most of the existing datasets reached saturation, since most of the results are in the range 95–99% of the perfect solution [22,23] Nevertheless, external non-technical challenges, such as lighting variations and weather condition changes, occlusions or damaged images, even variations in traffic signs among different countries, may decrease the system’s performance [17].

Thus, the last algorithms and the newest datasets are focused on the recognition under complex conditions. In this paper, we did a deep analysis for all publicly available datasets as of today, which is summarised in Table 1.

### 3.2. Ceit Traffic Sign Recognition Dataset

**Figure 1:**
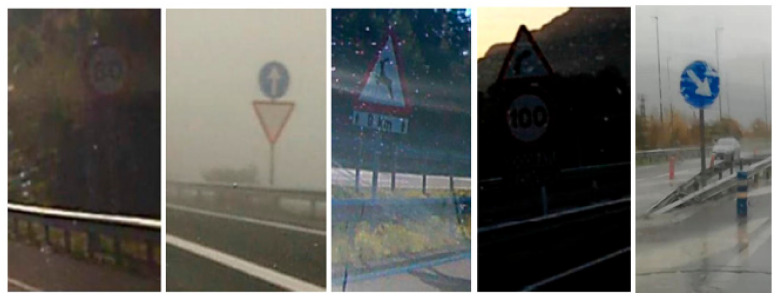
Ceit-TSR database. Sample images showing some of the challenging conditions: low contrast, fog, reflections, shadows and heavy rain.

Ceit-TSR consists of 264 colour images captured from 40 different videos of driving tracks within the Basque Country (Spain). They were recorded using different mobile phones and onboard cameras located on the dashboard. The images of this dataset were specifically selected so that, in addition to the different weather and light conditions that are covered in other existing datasets, they would also include other complex conditions. Those incorporate images with motion-blur, low-resolution signs, distant signs, low contrast and windshield artefacts (reflections, raindrops, dirtiness, etc.), as is shown in Figure 1. All images were manually annotated using the *Image Labeler* MATLAB tool, resulting in 418 bounding boxes that were also classified in six different categories: speed-limit, prohibitory, mandatory, caution, yield and restriction end. Finally, an extra category was added for the signs that did not fit the defined ones.

### 3.3. Existing Fog Detection Datasets

Although there are large-scale road datasets, such as KITTI [24], Cityscapes [25], Mapillary Vistas [26], ApolloScape [27] and BDD100k [28], the availability of useful image datasets for the foggy scenes evaluation is very low. Most of the existing datasets contain few or even no foggy scenes due to the difficulty of collecting and annotating them. For example, the Mapillary Vistas database contains 10 images out of 25,000 of misty images (not dense fog). Thus, some of the existing foggy datasets are generated from synthetic images or real-world images post-processed with synthetic fog (see Table 2 for a summary).

The Foggy Road Image Dataset (FRIDA) is the most popular one, which was created by Hautière et al. with synthetic images [29] and was later extended [30]. Among these images, six images of each scene show different kinds of fog: no fog, uniform fog, heterogeneous fog, cloudy fog, cloudy heterogeneous fog and finally a depth map image is also included.

A more recent dataset derived from the Cityscapes dataset was generated by Sakaridis et al., which is called Foggy Cityscapes [31]. It constitutes a collection of images from the original dataset that are processed and automatically annotated into foggy images using a fog simulator. Same authors also generated a new dataset with real-world foggy road scenes. The Foggy Driving dataset contains 101 light fog images captured with a cell phone camera at different points of Zurich and also with images collected from the web. Later on, they extended this dataset with the collected video frames of the same city and its suburbs improving the resolution and having much wider variety of scenes and different fog levels; this last dataset is named Foggy Zurich [32]. 

Finally, the SeeingThroughFog dataset [33] was developed in the context of the DENSE project. It records 10,000 km of driving in Northern Europe under different weather and illumination conditions in February and December 2019. The resulting annotations contain 5.5 k clear weather frames, 1 k captured in dense fog, 1 k captured in light fog and 4 k captured in snow/rain. 

### 3.4. Ceit Fog Detection Dataset

**Figure 2:**
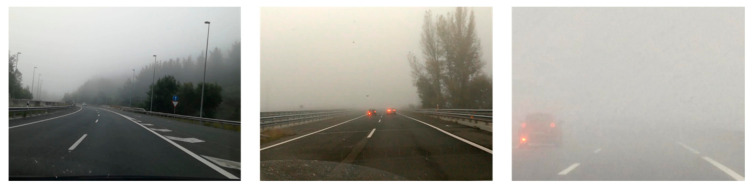
The Ceit-Foggy dataset. Sample images showing the annotated three different fog levels; from left to right: light fog, moderate fog and heavy fog.

The Ceit-Foggy dataset consists of a set of 41 videos corresponding to approximately 300 km of driving through the Basque Country and Segovia. These videos were recorded in different weather conditions, as shown in Figure 2. In total, 4480 frames have been extracted and labelled in five categories: sunny, cloudy, light fog, moderate fog and dense fog. Additionally, in some videos, if there was also rain or it was a dawn/dusk, this has also been specified. As for the Ceit-TSR dataset, these images were also recorded using different mobile phone and onboard cameras located on the dashboard. It is worth mentioning that these annotations just classify the general condition of the images; however, especially for long tracks, these conditions could change during the video.

## 4. Materials and Methods

### 4.1. Traffic Sign Recognition

**Figure 3:**
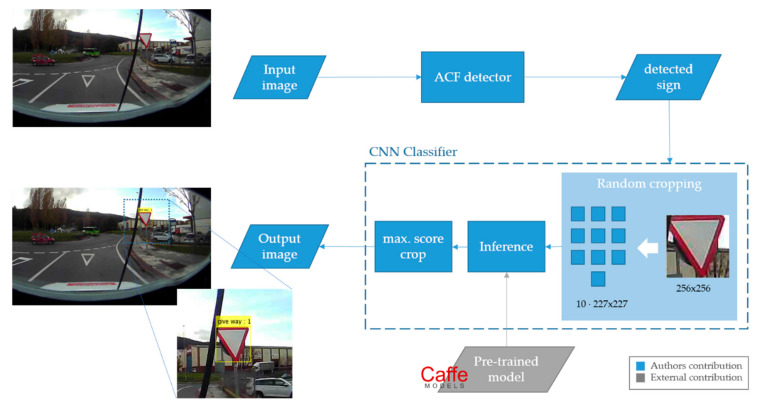
Methodology workflow implemented for the TSR module.

The implemented traffic sign recognition system is composed of two modules: detection and classification (see Figure 3). These modules were developed using MATLAB 2017a software. As it will be explained in the following sections, the authors did the fine-tuning of both algorithms, the detector and classifier, but not for the neural network used for the classification—the latter being a publicly available pre-trained model.

#### 4.1.1. Traffic Sign Detection

After the revision of the state-of-the-art of traffic sign detection methods, we concluded there is no clear framework that achieves the best results. Thus, we decided to implement and compare some of the most popular methods. Firstly, we tried using classic features, such as colour and shape, and modelling with the Viola Jones cascade detector. However, these alternatives were finally discarded as they were difficult to adjust and not very flexible with changing light conditions. 

**Figure 4:**
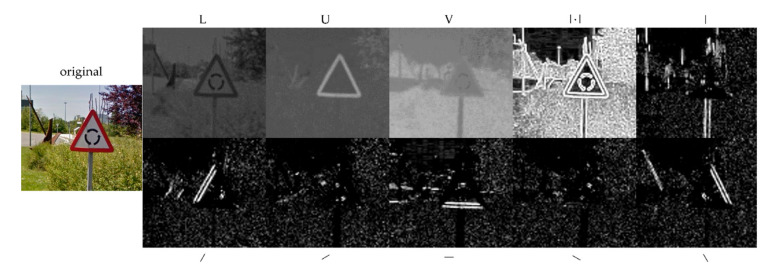
The Aggregated Channel Features (ACF). In the first row, from left to right: the original image, LUV channels, the gradient magnitude and individual representation of the HOG features at different angles of the sample sign.

Usually, existing detectors could be improved in two ways: using more complex features or implementing more powerful learning algorithms. Since the combination of boosting and cascading has proven to be very efficient for object detection [34], the key is to find representative characteristics at a low computational cost. In this context, a combination of record-breaking characteristics has emerged for pedestrian detection [35] and we tried to apply this method for traffic sign detection. The Aggregated Channel Features (ACF) detection framework uses an AdaBoost classifier trained with ACF features to classify image patches. The entire image is searched by using a multiscale sliding window approach. These ACF features consist of ten different channels: three from the LUV colour space, the gradient magnitude and the six oriented gradient maps (see Figure 4). Afterwards, the sum of every block of pixels of these channels is computed using fast features pyramids, and downscaled. Features are single-pixel lookups in the aggregated channels. Boosting is used to train and combine decision trees over these features (pixels) to accurately locate the object [35]. The channel extension offers a rich representation capacity, while the simplicity of the features allows a low computational cost. Therefore, the ACF detector was chosen to be implemented in this paper.

#### 4.1.2. Traffic Sign Classification

A pre-trained CNN model was used for the traffic sign classification, currently the most popular technique for object classification. This CNN model was trained from alexnet, applying transfer learning with the GTSRB dataset [36]. The authors of this model reported 97% accuracy in the first 50 GTSRB images. 

The input images were pre-processed before passing through the model to improve the efficiency of the CNN model. The pre-processing consists of the following minor changes: (1) Normalization of the image by subtracting the average image from the dataset. This is done to make the network less sensitive to differing background and lightening conditions. (2) Random cropping, since the alexnet requires input images of 227 × 227 pixels. For this aim, we decided to make 10 random crops of 227 × 227 on the 256 × 256 images. The 10 cropped images were classified and the bounding box with the resulting maximum score was selected. This way, the class with the highest confidence will be the final result of the classification.

### 4.2. Fog Detection

As it was explained in the state-of-the-art revision there are two main approaches for the detection of foggy scenes based on vision techniques: (1) measurement of the visibility range, and (2) extraction of image characteristics. The first approach was discarded for this application because there is no direct relation to the fog physical properties since several factors affect it, such as the background light, road curvature, presence of contrasted objects, etc. [37]. The high complexity of this problem could lead to the study of a solution with neural networks. However, this option was rejected given the hardware limitations as the application will run on an onboard system. So, the image feature extraction technique has been implemented. Although most of the previous works have analysed grayscale images, our work aims to study whether other colour spaces could help to get more information on the image and help to improve the results.

**Figure 5:**
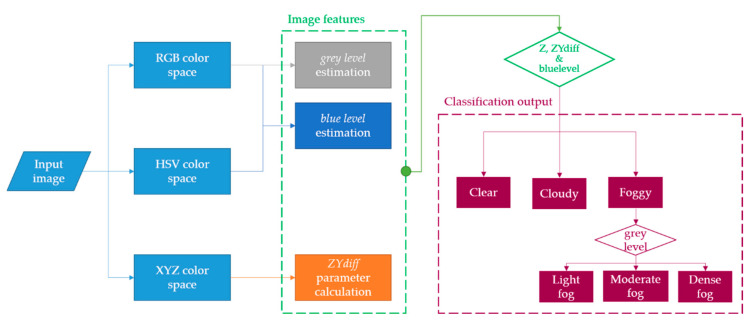
Methodology workflow implemented for the fog detection module.

After several experimental studies in the RGB and HSV colour spaces (Extra information about colour spaces is given at Appendix A), we concluded that this information was not sufficient to properly differentiate between cloudy and foggy scenes. Therefore, we appealed to a not so popular colour space, the XYZ 1. Hence, we designed a rule-based method from scratch that can classify sunny, cloudy and foggy scenes by using XYZ features. Afterwards, the designed algorithm estimates the fog level of the foggy images by using RGB and HSV features (see Figure 5).

#### Rule-Based Method

One of the main characteristics of fog is that it blocks visibility from a certain distance on. This causes a decrease in the contrast between the object and its background so that the scene takes on a white/grey colour.

Thus, our work extracts the specific features of the images and establishes several rules to classify scenes into sunny, cloudy and foggy. These rules are summarised in Table 3 and they will be presented in detail in the following lines. 

First, we analysed the XYZ colour space; here, the Z channel will find cloudy scenes and the parameter defined as *ZYdiff* will differentiate between foggy and sunny scenes. Second, once the foggy scene is detected, our algorithm will classify the foggy scenes into light-fog, moderate-fog and dense-fog, by using the RGB and HSV colour space-based features. The grey level will provide an estimation of how dense the fog is, and the blue level to calculate and confirm how clear the sky is. 

It is worth mentioning that, for these analyses, just pixels from the upper half of the image will be considered. In this portion of the image, we will find mostly the sky after having previously calibrated the camera position. 

*Z* and *ZYdiff* calculation

In the XYZ colour space, we have analysed the *Z* average level of the pixels located on the upper half of the image. We observed that this channel shows a difference between cloudy scenes and the rest, since the *Z* average value is lower on cloudy scenes (*Z* < 0.35). 

**Figure 6:**
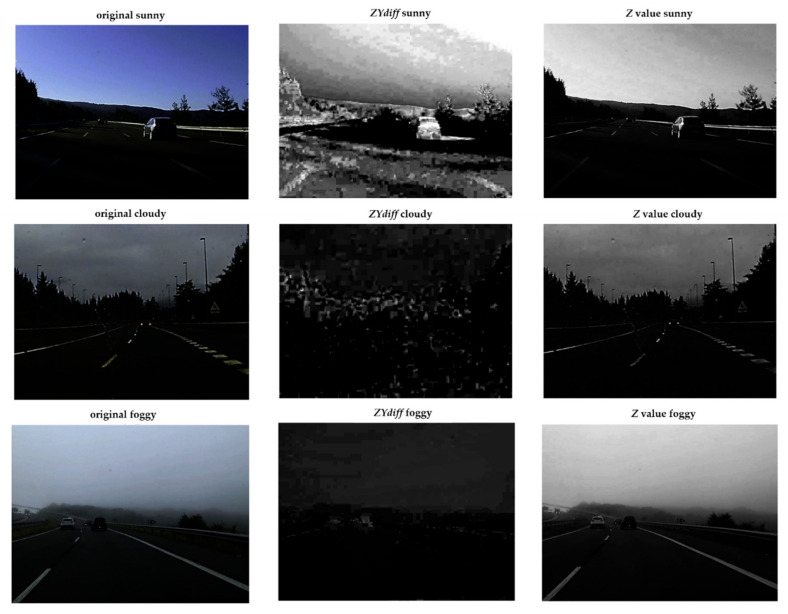
Studied features in three different weather scenes. From up to down—sunny, cloudy and foggy sample scenes.

However, this characteristic presents similar values both for sunny and foggy scenes. Therefore, a further feature is calculated by the $\frac{\left|Z-Y\right|}{Y}$ formula, which represents the difference of the *Z* and *Y* channels averages with respect to luminance (Y), hereinafter referred to as *ZYdiff.* This value is not relevant for cloudy situations but leads us to differentiate between sunny and foggy scenes, as can be seen in Figure 6. Thus, *ZYdiff* is higher on sunny scenes (*Z* > 0.1) than in foggy ones (*Z* < 0.1).

Grey level estimation

The grey level allows approximating how much contrast has been lost in the image due to fog. This feature has been extracted by establishing several rules to the RGB channels. Firstly, we considered as grey pixels those RGB values enclosed in the 140–255 range. This range represents bright pixel values that are not saturated. Additionally, we also limited the difference between each channel to 20; this rule will also ensure that the saturation of the pixel is low. 

Thus, this grey level would be the percentage of pixels that meet these conditions compared to the total number of pixels analysed on the upper half of the image.

The calculated *greylevel* seems to be a good representative of the fog level. Thus, based on experimental tests we define three thresholds that will conform the three different fog levels. This way, the light-fog scene is expected to have 20–30% of grey pixels, moderate-fog conditions will oscillate between 30 and 60% and an image with more than 60% of grey pixels will be considered a dense-fog scenario.

Blue level estimation

The same approach is followed for the calculation of the blue level; but, in this case, the HSV colour space is also introduced. This specific blue level was modelled by analysing portions of different images of clear sky that allows defining the following rules that combine both colour spaces:

For RGB colour space: B > G > R && (B-R) > 40.

For HSV colour space: 135 > H > 160 && S > 40 && V > 120.

Therefore, the blue level would be the percentage of pixels that meet these conditions compared to the total number of pixels analysed on the upper half of the image.

This *bluelevel* feature will help to confirm sunny scenarios classified by the *ZYdiff* parameter. 

In this manner, the fog detection matrix presented in Table 3 is finally constructed.

## 5. Results and Discussion

### 5.1. Traffic Sign Recognition Module

The ACF detector was taken as good for the target application. Additionally, a CNN-based classifier was also selected for the second phase of the recognition. Although, both the detector and classifier need to be validated for the specific implementation. 

For the evaluation of the behaviour of each of the implemented techniques, the models have been created using the GTSB training set. However, for the validation, the GTSB test set and the Ceit-TSR are used as the test images. 

#### 5.1.1. Detector Training

Since the learning process of an ACF detector is very similar to that of the cascade detector, this function also needs images with and without traffic signs. It uses as positive images all those that are passed as an argument while the negative ones are automatically generated.

The design and selection of the parameters for the detector are crucial to achieving the optimal implementation of it; thus, we did several fine-tuning experiments. We saw that

- the inference time was always about 200 ms per image;
- up to 5000 learners or decision trees do not improve the results;
- the object training size should be maximum 30 × 30 pixels, as bigger dimensions may increase the false negatives;
- a confidence threshold is key to compensate for false positives and negatives.

The final detector was trained with and object training size of 30 × 30 pixels. It used 5000 weak learners in 30 stages. It was tested with the GTSDB test set images, resulting in 100% precision and 76% recall; its inference time was 102 ms. On the other hand, this model achieves 96% precision and 68% recall when it is tested with the Ceit-TSR dataset. These means that the model experienced only a 4% precision decrease when dealing with detecting traffic signs in complex images (see Table 4). 

#### 5.1.2. Classifier Training

Concerning the classification of the detected signs, we implemented the CNN-based model that was trained with the GTSDB dataset. As was explained in Section 4.1.2, in this case, the model did not require any fine-tuning since it was pre-trained for this specific task, but we include additional pre-processing to the input images. 

Hence, to test the influence of the random cropping, we added it as a previous step before the classification. For this experiment, we used two different datasets: (1) 50 first images from GTSRB that are provided with the model and are already cropped with the ground-truthed ROIs; and (2) 50 first images from GTSRB that matches the (1) dataset, but with no crop applied—this is the original images from the dataset which retain some margin or background pixels in addition to the region of interest.

Thus, we see that the images that were already cropped to the ground-truthed ROI get better results. In this case, the random cropping has low influence since there was little room for improvement. However, on those input images that were not cropped, the pre-processing and random cropping leads to better results, as shown in Table 5. Therefore, it was decided to implement the methodology that includes the normalization of the original image by the subtraction of the mean image and the 10 random crops to later pass the resulting image to the classifier. It is considered that because the detector’s bounding box is sufficiently precise, the implemented classifier model achieves satisfactory results.

To validate this classifier, as was done on the detection phase, we used the test set of the GTSB dataset and Ceit-TSR dataset itself. Unlike the detector, the effect of the complex images that the Ceit-TSR dataset contains is notable in the classification phase (see Table 6). The maximum accuracy of 92% is achieved with the test images of GTSB but there is a 23% decrease when dealing with complex-condition images. 

**Figure 7:**
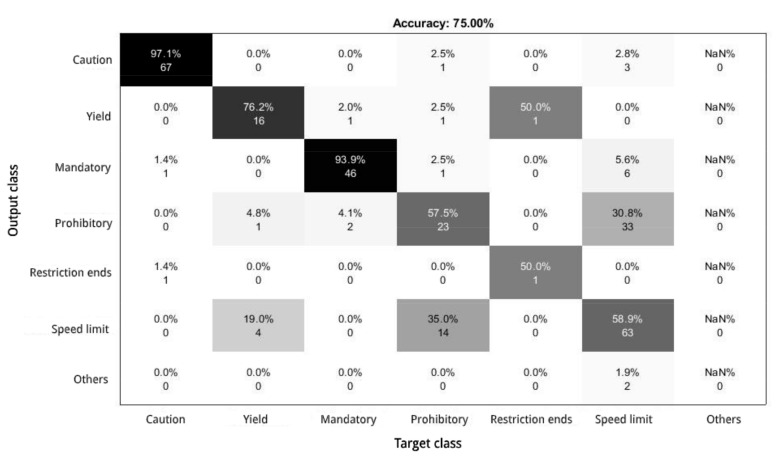
Confusion matrix of the final solution trained with the GTSB dataset and validated with the Ceit-TSR dataset.

This difference in results seems to be due to the fact that the classifier confuses some categories. As Figure 7 shows, even if the general accuracy was 75%, each sign category achieves a different score. For mandatory and caution signs the accuracy is more than 90% and the yield category scores a 76.2% accuracy. However, it is observed that the system is not capable of clearly distinguishing between the prohibitory and speed limit classes since its accuracy is less than 60%. This may be due to their similarity since both have the same geometry and colours. Besides, given the complexity of the images of the Ceit-TSR dataset, the information received from them may not have sufficient quality or details to detect their differences. Thus, this effect generates false positives and negatives in the classification, causing a decrease in the average accuracy. Concerning the analysis of the restriction ends category, there are no sufficient samples to draw conclusions.

### 5.2. Fog Detection Module

The proposal presented in this paper targets the fog detection task based on the XYZ, HSV and RGB colour spaces’ feature extraction. 

For the validation of this algorithm, we used the Ceit-Foggy dataset presented in Section 4.1.2. This dataset contains different scenes that allow us to validate whether our proposed algorithm can distinguish between sunny, cloudy and foggy scenes and in these last ones if we can estimate the fog level from 1–100. Additionally, due to the complexity of the real-world scenes, the proposed method also provides reliability data both for foggy and non-foggy scenes. Each event (fog/non-fog) will have the relative reliability data associated with it. The calculation of each reliability value is independent, and it is based on the repetitiveness of the data. Thus, when the fog status is stable the reliability will increase or decrease constantly and proportionally with the vehicle speed until the maximum (100) or minimum (0) is reached. However, if the fog status is oscillating, the abrupt changes will also lead to instability in the reliability of the data, which will not reach the extreme values. This way we can better detect and remove false detections that may occur due to isolated situations, such as white buildings, tunnels, veiled scenes, dirt on the lens, etc.

**Figure 8:**
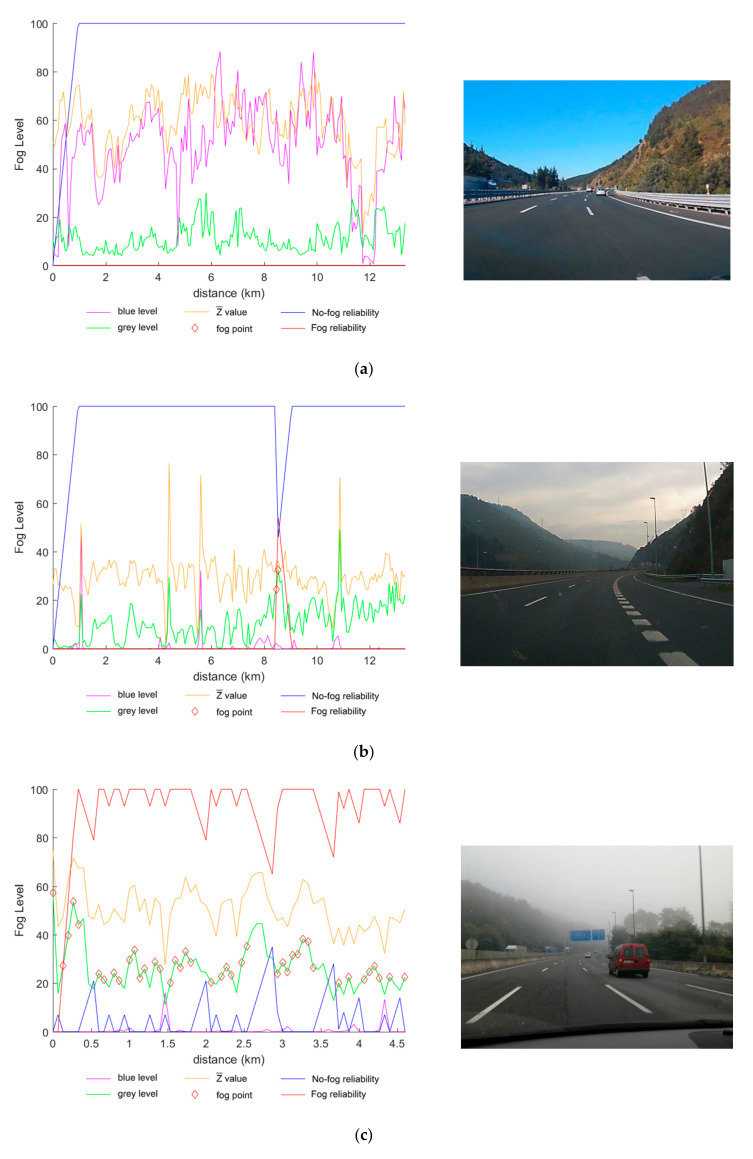

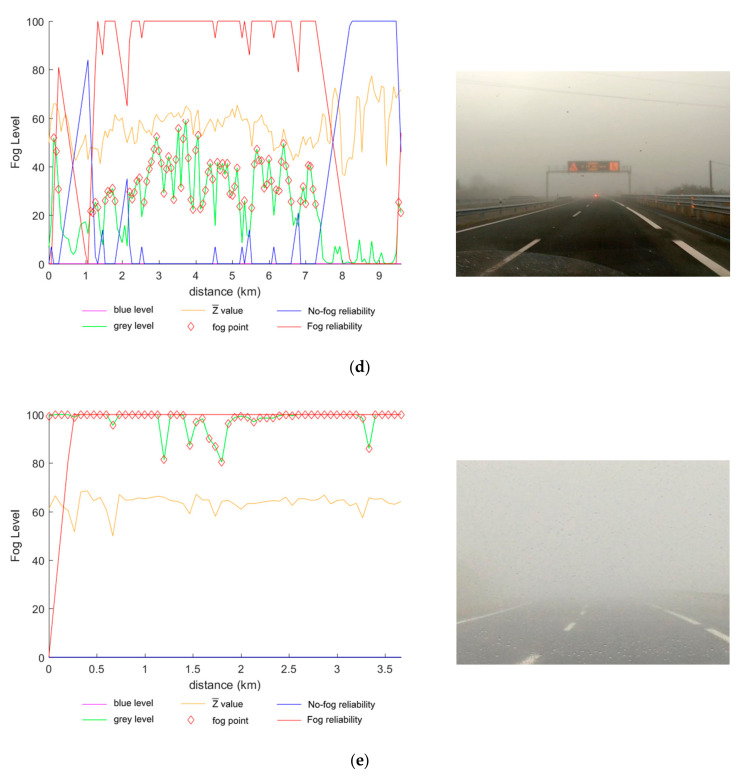
Extended offline analysis of five different videos. Each set of images presents a specific condi-tion: (**a**) sunny; (**b**) cloudy; (**c**) light-fog; (**d**) moderate-fog; and (**e**) dense-fog.

Figure 8 presents an extended offline analysis of the images extracted from five videos that were classified in each of the possible different scenes. These graphs show the grey level, $\bar{Z}$ value (multiplied by 100 for visualization purposes) and blue level that will guide the inspection of the scene type. Additionally, reliability data, both for fog and no-fog events, are drawn to discriminate false positives or false negatives. Finally, the frame that is classified as a foggy scene is also marked with a diamond. 

Figure 8a shows a sunny scene where there is no point marked as a foggy scene. $\bar{Z}$ is greater than the threshold of 0.35 and the blue level is also high while the grey level is below the limit of 20%. Thus, the reliability of no-fog is maximum once the algorithm has analysed the minimum number of images.

Figure 8b shows a cloudy scene where there are just two frames classified as foggy. In this case, the $\bar{Z}$ is the parameter that classifies the image as cloudy since its value is below the threshold of 0.35. It also can be observed that the blue levels are very low and grey levels are around 20%. These two false positives classified as foggy scenes at kilometre 8.5 could be easily discarded since the reliability is not high and it seems to be a punctual behaviour (≈200 m).

Figure 8c presents a light-fog scene video. As was expected, the $\bar{Z}$ value is above the 0.35 threshold and the grey level is in the range of 20–30%. Since the ranges set for light-fog are small, there are some fluctuations and therefore the reliability of the data is around 70–100%.

Figure 8d was labelled as a moderate-fog scene. This image set is a very clear example of a fog bank since at the start and end of the graph no fog is detected. During the fog bank, most of the points present a grey level value between 30 and 60%, as expected, and the $\bar{Z}$ value is also above the 0.35 limit. The reliability lines help also to confirm this fog-bank since the fog positive reliability (red line) is high during the fog period and then reversed with the non-fog reliability when leaving the fog bank. 

Figure 8e is the simplest case; the $\bar{Z}$ value is above 0.35, as expected, and the grey level is almost saturated due to the dense fog of the scene. Consequently, all points are marked as positives and the fog reliability is 100%. 

## 6. Conclusions

In this paper, two vision-based ITS applications are proposed, one for traffic sign recognition and one for fog detection that will be running in real-time for an onboard probe vehicle system. Traffic sign detection is covered with an ACF detector whereas the classification phase was approached by a CNN, achieving an overall accuracy of 92%. Fog detection instead is carried out using classic computer vision methods by image feature extraction through a combination of RGB, HSV and XYZ colour spaces. The achieved inference time in both applications allows us to make a real-time analysis for an onboard system with a performance close to the reviewed state-of-the-art methods. This real-time analysis makes it possible to convert these implementations into cooperative services that will not only support the driver but also give valuable information for other road users. 

These two C-ITS modules are already integrated on an onboard barebone industrial mini PC and are being validated on-site. In the case of TSR application, the presented module supports the operators in charge of the road maintenance to create an exhaustive traffic signal inventory and detect possible deficiencies that may require the attention of an expert. Besides, the fog detection module sends weather-related events to the server back-office and ITS-G5 beacons for communication with other vehicles. The generation of this real-time traffic alerts could enhance road safety by warning the drivers of road hazards, such as low visibility due to fog banks. 

## 7. Future Research Direction

As future work, the CEIT’s Sustainable Transportation and Mobility group is working on an extension of the TSR module for the inspection of road markings. Good preservation of road markings is vital for road safety and becomes even more important for the integration of future self-driving cars. Thus, this additional functionality would allow us to study the status of the paint and advise the road maintenance operators to take specific actions before the degradation is safety-critical. 

The integration of new modules, or the extension of them, will probably require improving the computing capacity of the onboard system. To this end, it is possible to explore the integration of 5G technology into the system, which that would allow the images to be sent to external high-performance computers and return the result with minimal latency.

If there were no hardware limitations, new algorithms based on deep learning could be explored for further improvements on fog detection. Moreover, the current rain module of the system, which is based on an IR sensor, could also be enhanced by data fusion with computer vision techniques. For example, a detector of the splash effect of the vehicle’s wheels when there is a water film accumulated on the asphalt. 

Besides, further improvements could be done to the overall system by designing an interface for the visualization of the C-ITS events by using an HMI solution that could go from simply using a mobile application to augmented reality options on the windscreen. 

## Figures and Tables

**Table 1:** Summary of the existing databases for traffic sign recognition (TSR) and its principal characteristics. It is also included our database Ceit-TSR.

	Purpose	Number of Images	Number Bounding Boxes	Classes	Categories	Resolution	Videos	Challenging Conditions	Country	Publication Year
GTSDB	Detection	900	1213	43	4	1360 × 1024	no	no	Germany	2011
GTSRB	Classification	51,840	1728	43	4	1360 × 1025	no	no	Germany	2013
Extended-GTSDB	Detection/Classification	900	2655	164	8	1360 × 1024	no	no	Germany	2020
BTSD	Detection	25,634 (9006 annotated)	13,444	--	--	1628 × 1236	yes (4)	no	Belgium	2011
BTSC	Classification	7125	--	62	--	1628 × 1237	yes	no	Belgium	2011
TT100K	Detection/Classification	100,000	30,000	45	--	2048 × 2048	no	no	China	2016
STS	Detection/Classification	20,000	3488	7	--	1280 × 960	no	no	Sweden	2011
RSTD	Detection/Classification	179,138	15,630	156	6	1280 × 720 1920 × 1080	no	no	Russia	2016
LISA	Detection/Classification	6610	7855	49		640 × 480–1024 × 522	yes (17)	no	USA	2012
Stereopolois	Detection/Classification	847	251	--	10	960 × 1080	no	no	France	2010
MASTIF	Detection/Classification	4875	13,036	--	5	720 × 576	yes	no	Croatia	2011
CTSD (Chinese)	Detection/Classification	1110	1574	48	--	1024 × 768 and 1270 × 800	no	no	China	2016
CCTSD	Detection/Classification	10,000	13,361	--	3	several	no	no	China	2017
ETSDB	Detection/Classification	82,476	--	164	4	several	no	yes	Belgium, Croatia, France, Germany, Netherlands, Sweden	2018
DITS (1)	Detection	1887	--	--	3	1280 × 720	no	yes	Italy	2016
DITS (2)	Classification	9254	--	58		1280 × 721	no	yes	Italy	2016
KTSD	Detection	498	832	--	3	several	no	yes	Korea	2017
CTSD (Complex)	Detection/Classification	2205	3755	153	3	several	no	yes	China	2018
Cure TSD	Detection/Classification	896,700	648,186	14	--	1628 × 1236	yes (2989)	yes	Belgium	2020
Ceit-TSR	Detection/Classification	264	418	--	6	several	no	yes	Spain	2020

**Table 2:** Summary of the existing datasets for fog detection and its principal characteristics.

	Number of Images	Classes	Resolution	Country	Synthetic	Publication Year
FRIDA1	90	--	640 × 480	--	yes	2010
FRIDA2	330	--	640 × 480	--	yes	2012
Foggy Cityscapes-coarse-	20,000	8	2040 × 1016	Germany and Switzerland	synthetic fog	2018
Foggy Cityscapes-refined-	550	8	2040 × 1016	Germany and Switzerland	synthetic fog	2018
Foggy Driving	101	19	960 × 1280	Zurich	no	2018
Foggy Zurich	3808 (40 annotated)	19	1920 × 1080	Zurich	no	2019
Seeing Through Fog	1,429,060	--	1920 × 1024	Germany, Sweden, Denmark, and Finland	no	2020
Ceit-Foggy	4480	--	several	Spain	no	2020

**Table 3:** The proposed rule-based method for fog detection and fog level estimation. This method anal-yses the RGB, HSV and XYZ colour spaces.

Sunny	*Z* > 0.35 && *ZYdiff* > 0.1 *bluelevel* > 0.3		
Cloudy	*Z* < 0,35		
Foggy	*Z* > 0.35 && *ZYdiff* < 0.1	Light	*greylevel* (20–30)
		Moderate	*greylevel* (30–60)
		Dense	*greylevel* (60–100)

**Table 4:** Summary of detection results for the GTSB test set and Ceit-TSR dataset. Complex images made the model decrease its precision by 4% compared with the GTSB result.

Test DB	Precision	Recall	F-Score	Inference Time (ms)
GTSB (300 images)	1	0.76	0.864	102
Ceit-TSR (264 images)	0.96	0.68	0.796	118

**Table 5:** Comparison of the random cropping influence on the classification result as a pre-processing technique.

Pre-Processing Technique	Test Images	Accuracy	Inference Time (ms)
image normalization + 10 random cropping	50 images ROI cropped	0.98	40
	50 original images	0.76	42
image normalization + 227 × 227 re-scaling	50 images ROI cropped	0.96	18
	50 original images	0.52	22

**Table 6:** Summary results of the classification for the GTSB and Ceit-TSR datasets. The influence of the complex images on the selected classifier is notable.

Test DB	Accuracy	Inference Time (ms)
GTSB (300 images)	0.92	43
Ceit-TSR (264 images)	0.75	43

**Figure A1:**
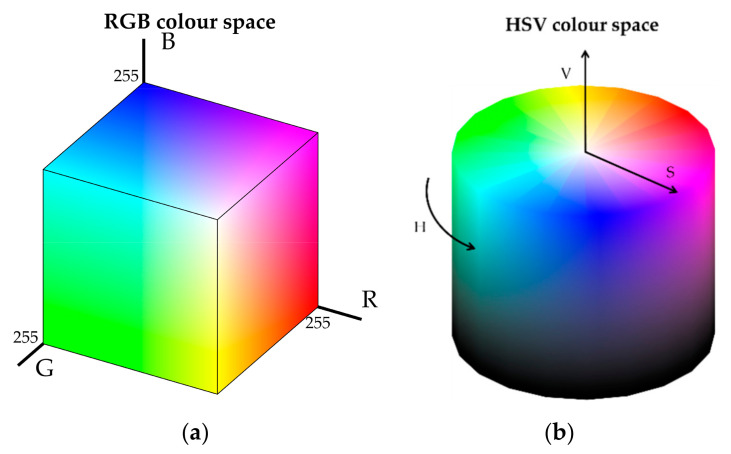
Representation of the (**a**) RGB and (**b**) HSV colour spaces.

**Figure A2:**
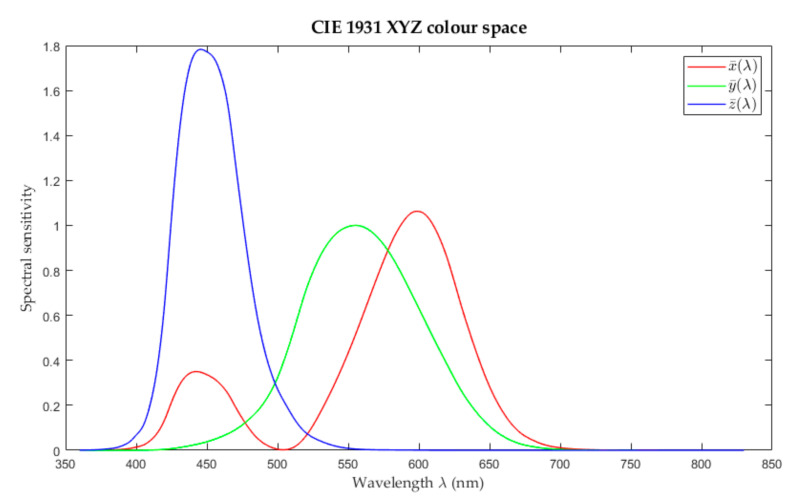
The CIE XYZ standard observer colour matching functions. Adapted from Jeff Mather (2021). Spectral and XYZ Color Functions (https://www.mathworks.com/matlabcentral/fileexchange/7021-spectral-and-xyz-color-functions. Accessed on 15 January 2021) MATLAB Central Exchange Retrieved 5 January 2021.

## Data Availability

Ceit-TSR and Ceit-Foggy datasets are available online at https://github.com/oipa/CITS-traffic-alerts (accessed on 9 Febuary 2021).

## Appendix A

In this section, there is a brief description of the three colour spaces that have been used in this work to extract characteristics from the image.

### Appendix A.1. RGB Colour Space

This colour space is the most common one since the human eye only has colour sensitive receptors for red (R), green (G) and blue (B). Thus, it is theoretically possible to decompose every visible colour into combinations of these three “primary colours” with different range of intensities from 0 to 255. Thus, this combination can be represented as a three dimensional coordinate plane with the values for R, G and B on each axis (see Figure A1a). This way we can conclude that when all channels have a value of zero, no light is emitted resulting on black colour. Whereas when all three colour channels are set to their maximum the resulting colour is white.

### Appendix A.2. HSV Colour Space

HSV colour space is a cylindrical representation where colours of each hue (H) are arranged in a radial slice (see Figure A1b). The hue is the colour portion of the model that is expressed in 0–360 degrees where it can be considered colours like red, yellow, green, cyan, blue and magenta each in 60-degree increments. The central axis represents the value (V) or brightness of the colour from 0 to 100 percent, where 0 is completely black and 100 is the brightest and reveals the most colour. Finally, saturation (S) describes the amount of grey in a particular colour; its minimum value (0) introduces more grey and the maximum (100) is the most similar to its primary colour.

### Appendix A.3. XYZ Colour Space

The CIE XYZ colour space encloses all colour sensations that are visible to the human eye. The human eye with normal vision has three kinds of cone cell that sense light, S, M and L, depending on their sensitivity to short-, middle- or long-wavelength light. The CIE colour model takes the luminance (as a measure for perceived brightness) as one of the three colour coordinates, calling it Y. The spectral response of the luminance is specified as the photopic luminosity function. The maximum possible Y value, e.g., for a colour image, may be chosen to be 1 or 100, for example. The Z coordinate responds mostly to shorter-wavelength light (blue colours on the visible light spectrum), while X responds both to shorter- and longer-wavelength light [38]. Figure A2 shows the used colour matching functions.
